# Pharmacological Activity of *Garcinia indica* (Kokum): An Updated Review

**DOI:** 10.3390/ph14121338

**Published:** 2021-12-20

**Authors:** Sung Ho Lim, Ho Seon Lee, Chang Hoon Lee, Chang-Ik Choi

**Affiliations:** 1 Integrated Research Institute for Drug Development, College of Pharmacy, Dongguk University-Seoul, Goyang 10326, Korea; 93sho617@naver.com (S.H.L.); ghtjsrhtn@naver.com (H.S.L.); 2 BK21 FOUR Team and Integrated Research Institute for Drug Development, College of Pharmacy, Dongguk University-Seoul, Goyang 10326, Korea; uatheone@dongguk.edu

**Keywords:** *Garcinia indica*, kokum, wild mangosteen, pharmacological activity

## Abstract

*Garcinia indica* (commonly known as kokum), belonging to the Clusiaceae family (mangosteen family), is a tropical evergreen tree distributed in certain regions of India. It has been used in culinary and industrial applications for a variety of purposes, including acidulant in curries, pickles, health drinks, wine, and butter. In particular, *G. indica* has been used in traditional medicine to treat inflammation, dermatitis, and diarrhea, and to promote digestion. According to several studies, various phytochemicals such as garcinol, hydroxycitric acid (HCA), cyanidin-3-sambubioside, and cyanidin-3-glucoside were isolated from *G. indica*, and their pharmacological activities were published. This review highlights recent updates on the various pharmacological activities of *G. indica*. These studies reported that *G. indica* has antioxidant, anti-obesity, anti-arthritic, anti-inflammatory, antibacterial, hepatoprotective, cardioprotective, antidepressant and anxiolytic effects both in vitro and in vivo. These findings, together with previously published reports of pharmacological activity of various components isolated from *G. indica*, suggest its potential as a promising therapeutic agent to prevent various diseases.

## 1. Introduction

The use of medicinal herbs as medicine is the oldest form of medical treatment known to humanity and has been used in all cultures throughout history [1]. Since time immemorial, humans have recognized their dependence on nature for healthy living, and have relied on a variety of plant resources for medicine to cure numerous diseases [2]. This indigenous knowledge, passed down from generation to generation in different parts of the world, has contributed significantly to the development of traditional medical systems [3], as well as provided a scientific basis for their traditional uses by exploring various biologically active natural products [4]. For instance, between 1981 and 2014, about 26% of new chemical entities were natural products or derived from natural products [5]. They are widely used in the prevention and treatment of clinical diseases as they have the unique advantages of low toxicity and side effects compared with chemical drugs [6]. Among medicinal plants, Clusiaceae contains approximately 50 genera and 600 species [7], and has been extensively used in ethnomedicine to treat a number of disease conditions, including wounds, ulcers, dysentery, cancer, inflammation, and infection [8,9].

**Figure 1:**
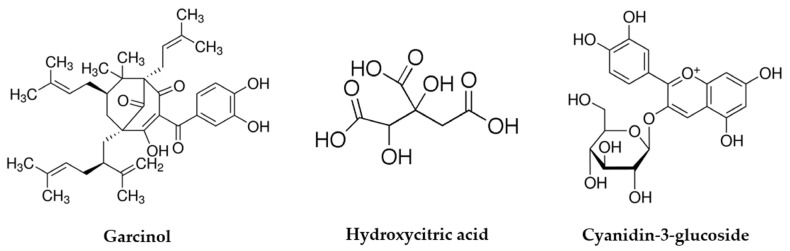
Chemical structure of bioactive ingredients isolated from *G. indica*.

*Garcinia* belongs to the Clusiaceae family (Mangosteen family) and has multiple application in the culinary, pharmaceutical, and industrial fields [10]. The plants are distributed around the world including tropical Asia, Africa, and Western Polynesia [11]. In the last few decades, they have received considerable attention and extracts of different plant parts of the Garcinia species, e.g., *Garcinia brasiliensis*, *G. cambogia*, *G. gardneriana*, *G. pedunculata*, and *G. mangstana* have demonstrated potential effectiveness in the prevention and treatment of non-transmissible chronic diseases [12]. Furthermore, it was reported that they contain a wide range of biologically active metabolites and that the chemical compositions of their extracts are rich in bioactive molecules including hydroxycitric acid (HCA), bioflavonoids, procyanidines and polyisoprenylated benzophenone derivatives including garcinol, xanthochymol and guttiferone isoforms [9,13]. These compounds have been implicated in biological activities such as antioxidant [14], anticancer [15,16], and antiviral effects [17]. In particular, the major bioactive ingredients such as garcinol, HCA, and cyanidin-3-glucoside have been isolated (Figure 1), characterized and evaluated for their therapeutic properties. For example, garcinol showed anti-inflammatory effects by regulating various signaling pathways, molecular binding, and gene expression contributing to inflammation, such as inhibition of the cyclooxygenase-2, 5-lipoxygenase and inducible nitric oxide synthase synthesis [18,19,20]. Also, garcinol was reported to have antioxidant effects through high free-radical, superoxide anion (O^2-^) scavenging activity [21,22], and to have neuroprotective properties by functioning as a histone acetyltransferase inhibitor (HAT) [23]. In addition, recent studies demonstrated its anticancer effects by inducing apoptosis and cell cycle arrest, inhibiting of angiogenesis, and regulating of gene expression in oncogenic cells [24].

*Garcinia indica* is a plant native to certain regions of India [25]. It is an underexploited slender evergreen tree and is known as wild mangosteen, kokum, and goa butter tree [26]. All parts of *G. indica*, i.e., fruits, rind, seeds, etc., have been used in various culinary, industrial and pharmaceutical applications, as well as fruit drinks and food [27]. Its pharmacological properties including antioxidant [28], anti-inflammatory activity [29], antimicrobial [30], anticancer [31], and anti-obesity [32] effects have been reported. The present review aims to discuss these recent studies and update the pharmacological properties of *G. indica*.

## 2. Methodology

We collected scientific literature on the origin, medicinal uses, and pharmacological activity of *G. indica* published in the English language up till 2021 from PubMed, Google Scholar, and Web of Science. The search terms were “*Garcinia indica*”, “kokum”, “pharmacological activity”, “antioxidant”, “anti-obesity”, “anti-inflammatory”, and “anti-diabetic”. 

As a result of the literature research, various pharmacological activities of *G. indica* were reported and reviewed [33,34,35], and a total of 7 papers were additionally reported recently. We comprehensively reviewed and updated the study design, results, and interpretation of each paper.

## 3. Pharmacological Properties of *G. indica*

A summary of the pharmacological properties of *G. indica* is described in Table 1.

### 3.1. Antioxidant Effects

The overproduction of oxidants can cause oxidative damage to biomolecules such as lipids, DNA, and proteins, increasing the risk of cancer, and cardiovascular and other diseases [43,44]. Antioxidants may reduce oxidative stress through various mechanisms [45]. In the past few years, there have been many reports on the antioxidant activity of *G. indica* [46,47,48,49,50,51,52,53]. Panda V et al. [36] reported the antioxidant effect of aqueous extracts of *G. indica* in an animal model of oxidative stress induced with ethanol (EtOH). Malondialdehyde (MDA), a lipid peroxidation marker, was elevated in the EtOH-treated group compared with the normal group. Treatment with the aqueous extracts of *G. indica* reversed these results. The reduced glutathione (GSH) level in the EtOH-treated group was significantly reduced compared with the normal group (*p* < 0.001). Treatment of the EtOH-treated group with aqueous extracts of *G. indica* (400 and 800 mg/kg) significantly restored the decreased levels of GSH (*p* < 0.05 and *p* < 0.01, respectively). In addition, superoxide dismutase (SOD) and catalase (CAT) activities were also markedly lower in the EtOH-treated group compared with the normal group (*p* < 0.01). The reduction in SOD and CAT activity levels were improved in a dose-dependent manner when treated with the aqueous extracts of *G. indica*. In particular, the high-dose group treated with 800 mg/kg of aqueous extracts of *G. indica* recovered to near normal levels. Furthermore, treatment with aqueous extracts of *G. indica* significantly ameliorated glutathione peroxidase and glutathione reductase activity, which were decreased by EtOH treatment (*p* < 0.01). 

Nanobiotechnology is a new field of research related to the convergence of biology and nanotechnology [54]. A recent study successfully demonstrated the use of plant extracts for the biogenic synthesis of silver nanoparticles (AgNPs) with antioxidant activity [55]. However, the synthesis of AgNPs was mainly achieved by physical and chemical methods of metal nanoparticle synthesis, which includes the use of hazardous chemicals that limit their potential use in biomedical applications [56]. To overcome these disadvantages, biosynthesis using various biological agents such as bacteria, fungi, plants, and their extracts is a green chemistry approach that is inexpensive, does not use hazardous chemicals, and has excellent biocompatibility and low toxicity [57]. Sangaonkar, G.M. et al. [37] optimized parameters for a simple, stable and benign biosynthesis method of AgNPs using *G. indica* fruit extract. Then, they performed 1,1-diphenyl-2-picrylhydrazyl (DPPH) free-radical scavenging, reducing power activity, and hydrogen peroxide and nitric oxide (NO) radical scavenging activity to evaluate the potential antioxidant activity of AgNPs biologically synthesized with *G. indica* fruit extract. They found that the tested range of AgNPs (20, 40, 60, 80, and 100 µg/mL) inhibited DPPH activity in a dose-dependent manner and exhibited a 57% inhibitory effect at the maximum concentration. Similar to the DPPH activity, the NO radical scavenging activity of AgNPs was confirmed to be dose-dependent, but lower compared with the DPPH activity. Furthermore, A 100 μg/mL AgNPs, which was less than half of the dose of butylated hydroxytoluene used as a positive control, resulted in 27% NO radical scavenging. Similar to DPPH and NO radical scavenging activity, the inhibitory response of biological AgNPs to reducing power was increased in a dose-dependent manner. Whereas, as a result of H_2_O_2_ radical scavenging activity, the inhibitory activity of AgNPs at the highest concentration was 65%, which was similar to the inhibitory activity of 72% using the same test concentration of standard ascorbic acid. In conclusion, *G. indica* fruit extract containing significant amounts of natural antioxidants such as HCA can be used in environmentally friendly AgNPs, preventing the use of toxic chemicals. Therefore, *G. indica* has important applications in biomedical fields as a good source of reducing and capping agents.

Barve, K. [38] performed lipid peroxide, GSH, CAT, and SOD assays using C57BL/6 male mice to measure the antioxidant activity of the garcinol-enriched fraction (GEF). This fraction was prepared from the hexane extract of *G. indica*. The extracts were loaded on a silica column, and hexane and ethyl acetate were eluted in ascending order of polarity to obtain various fractions by flash chromatography at a flow rate of 15 mL/min. The collected fractions (12 mL) were confirmed for the presence of garcinol by thin-layer chromatography, and this flash chromatography procedure was repeated 3 times to obtain sufficient GEF. Lipid peroxidation and changes in the oxido-redox state were measured for enzymatic and non-enzymatic markers. Lipid peroxide, an indicator of oxidative stress, was twice as high in disease-induced animals fed a modified Western diet compared with normal animals (*p* < 0.001). Simvastatin treatment was used as a positive control and animals treated with GEF (50 and 100 mg/kg) showed significantly reduced effects (*p* < 0.05 and *p* < 0.01, respectively), although they were higher than those in normal animals. GSH levels in disease-induced animals were significantly depleted compared with those in normal animals (*p* < 0.001). Treatment with GEF (25, 50, and 100 mg/kg) strikingly ameliorated depleted GSH levels in a dose-dependent manner, although this did not reach normal levels. CAT and SOD activity levels were significantly reduced in disease-induced animals (*p* < 0.001 and *p* < 0.0001, respectively). GEF treatment at 25 mg/kg reversed CAT activity levels, but this did not reach statistical significance. However, GEF treatment at 50 and 100 mg/kg significantly restored the CAT activity levels to levels similar to those of normal animals. Of note, only 100 mg/kg GEF treatment significantly increased the SOD activity level. 

In another study conducted by Patel et al. [39], the IC_50_ value for the DPPH scavenging activity of an aqueous extract of *G. indica* was estimated to be 231.85 ± 21.56 μg/mL. They also used animal models to measure biomarkers related to oxidative stress, including SOD, CAT, and thiobarbituric acid reactive substance (TBARS). The group treated with 85 mg/kg of isoprenaline (ISO) showed significantly reduced SOD and CAT values (5.38 ± 0.31 unit/g of tissue and 0.54 ± 0.02 µmol of H_2_O_2_ consumed/min/g of tissue, respectively) compared to the normal group (13.55 ± 0.62 unit/g of tissue and 2.66 ± 0.24 µmol of H_2_O_2_ consumed/min/g of tissue, respectively). Treatment of the ISO-induced toxicity group with an aqueous extract of *G. indica* at 250 and 500 mg/kg slightly restored the SOD levels (6.12 ± 0.93 and 7.56 ± 0.22 unit/g of tissue, respectively) and slightly improved the CAT levels (1.1 ± 0.17 and 1.08 ± 0.19 µmol of H_2_O_2_ consumed/min/g of tissue, respectively). Furthermore, treatment with ISO increased the TBARS level (3.82 ± 0.21 nmol MDA/g of tissue) compared with the normal group (1.65 ± 0.12 nmol MDA/g of tissue). The tested concentrations of aqueous extracts of *G. indica* did not affect these values (2.55 ± 0.25 and 2.17 ± 0.13 nmol MDA/g of tissue, respectively) and treatment of the normal control group with 500 mg/kg *G. indica* aqueous extract did not change the levels of any of the three biomarkers related to oxidative stress. They used the lower dose range because the antioxidant enzyme levels were not restored in the 250 and 500 mg/kg *G. indica*-treated groups.

Dhamija I et al. [40] measured antioxidant activity to elucidate the mechanism of antidepressant effect in various animal models. *G. indica* at 1% *w*/*w* significantly decreased monoamine oxidase levels (MAO-A and MAO-B) in brain homogenates, similar to a group treated with fluoxetine. By measuring the MDA concentration in the brain homogenate, a direct correlation was observed between the dose of *G. indica* and a decrease in the MDA level, suggesting a decrease in free-radical production.

### 3.2. Anti-Obesity Effects

Recently, as the prevalence of overweight and obesity has reached epidemic levels, the incidence of various comorbidities such as type 2 diabetes, cardiovascular disease, and cancer has rapidly increased, causing enormous health and economic burdens [58,59]. Medicines and bariatric surgery are the main strategies to prevent and treat obesity, but there are side effects [60]. For this reason, various natural products and their components are being tested to improve obesity with minimal side effects [61]. An anti-obesity effect of garcinol isolated from *G. indica* was reported [62,63,64]. 

As mentioned above, Barve, K. [38] confirmed the reduction of the oxidative stress response of GEF. In addition, he conducted research into the anti-obesity effects of GEF. C57BL/6 male mice were randomly divided into 6 groups of 6 mice each: Group 1 (normal control); Group 2 (disease-induced control by a modified Western diet); Group 3 (positive control receiving 8 mg/kg of simvastatin); and Groups 4, 5, and 6 (treated with 25, 50, and 100 mg/kg of GEF). Compared with Group 1, the weights of animals in all groups were significantly increased at 12 weeks. Group 2 weights continued to increase significantly until 16 weeks (*p* < 0.05), but those in Groups 3, 4, 5, and 6 treated with simvastatin and GEF decreased significantly from 12 to 16 weeks (*p* < 0.001 and *p* < 0.0001, respectively). Group 2 showed significant negative changes in the lipid profile indicative of disease induction. Groups 3, 4, 5, and 6 showed a decrease in the increased total cholesterol and triglyceride levels induced by a Western diet in a concentration-dependent manner, In particular, the values of Group 6 were similar to those of Group 1 (*p* < 0.01 and *p* < 0.0001, respectively). Furthermore, treatment with different concentrations of GEF elevated high-density cholesterol levels in a concentration-dependent manner and significantly decreased low density cholesterol levels to those of positive control levels (*p* < 0.0001).

Tung YC et al. [41] investigated the potential anti-obesity effects of *G. indica* fruit extract (GIE) in a 3T3-L1 cell model and high-fat diet (HFD)-induced obese animal model. First, 3T3-L1 preadipocytes were used for the following experiments after differentiation induced by differentiation medium (DMI) containing 5 μg/mL insulin, 0.5 mM 3-isobutylmethylxanthine, 1 μM dexamethasone, and 2 μM rosiglitazone. After confirming the lipid content by Oil Red O staining, the groups treated with 1 and 2 μg/mL GIE showed significantly reduced triglyceride levels to 63.5% and 65.6% without cytotoxic effects, compared with the group treated with only DMI. However, treatment with GIE in the tested range did not affect the amount of glycerol in the medium. The weights of C57BL/6 male mice fed a normal diet (ND) or HFD for 12 weeks were 27.5 and 37.4 g, respectively. The body weights of the group supplemented with 0.01% GIE in HFD were significantly reduced to 34.4 g (*p* < 0.05), and no effects on liver, kidney, or spleen weight were observed. A significantly reduced weight of mesenteric, perigonadal and retroperitoneal fat was noted in the group supplemented with GIE. Treatment with GIE also reduced the ratio of body fat compared with the HFD group. In addition, the histopathological examination of liver and the perigonadal adipose tissue of mice demonstrated that GIE treatment reduces the adipocyte size and liver fat accumulation. They further investigated the expressions of proteins related to adipogenesis, lipolysis and β-oxidation to elucidate the mechanisms involved in these effects. There was no significant difference in PPARγ and C/EBPα, proteins related to adipogenesis, between the ND and HFD groups, whereas PPARγ and C/EBPα protein levels were significantly decreased after supplementation with GIE compared with the HFD group. Although there was no significant difference in the protein levels of CPT-1A between the ND and HFD groups, supplementation with GIE was significantly elevated in the protein levels of CPT-1A compared to the HFD group. Moreover, a significant increase in PPARα protein levels was observed in the GIE supplemented group compared with the HFD group. These results suggest that GIE exerts anti-obesity effects in 3T3-L1 adipocytes and animal models by inhibiting adipogenesis and increasing β-oxidation.

### 3.3. Anti-Arthritic Activity

Arthritis is highly prevalent worldwide and can be divided into types, the most common of which are rheumatoid arthritis, osteoarthritis, psoriatic arthritis and inflammatory arthritis [65]. The exact cause of arthritis is not yet clear, and although it is commonly used to treat various types of anti-inflammatory arthritis, it is associated with serious side effects such as gastric bleeding and an increased risk of other cardiovascular problems [66]. Consequently, it is very important to explore effective and safe anti-arthritis drug candidates isolated from natural products.

Warriar, P. et al. [42] evaluated the anti-arthritic effects of GEF in rheumatoid arthritis. An extract (9 gm) of *G. indica* was adsorbed onto silica and loaded. Thereafter, fractions were obtained through stepwise gradient elution with n-hexane-ethyl acetate, including a fraction (800 mg) showing the maximum concentration of garcinol. A rat model of adjuvant arthritis (AA) was developed with a single injection of 0.1 mL of Complete Freund’s Adjuvant (CFA). Rats were divided into 4 groups of 6 each as follows: Group 1 (normal control); Group 2 (disease control of CFA alone); Group 3 (positive control receiving 10 mg/kg of diclofenac sodium); Group 4 (treated with 10 mg/kg of GEF). To examine the major lesions and determine the effects of the therapeutic agents, the volume of the left hind paw of rats was measured on days 0, 1, 5, 12, 16, and 21 using an electronic transfusion meter. To determine the severity of the secondary lesions, the volume of the right hind paw was also measured. In addition, to evaluate the severity of AA, the ears, nose, tail, forepaws, and hind paws of the rats were visually examined for inflammatory lesions, and arthritis severity was scored according to the redness, swelling, and presence of nodules [67]. The CFA-treated group developed primary arthritis in the left hind paw, and compared with the normal group, a significant level of swelling was observed and maintained for 21 days (*p* < 0.0001). Treatment with GEF significantly suppressed paw swelling from days 5 to 21, similar to the positive control group, when compared with the disease control group. On the other hand, the GEF-treated group also had a decrease in the swelling of the right hind paw, but this was not statistically significant. CFA-treated rats showed a significant increase in the arthritis index compared with the control group, reaching a maximum at 5 days, and then gradually decreasing. The groups treated with 10 mg/mL of GEF and diclofenac had a significantly reduced arthritis index compared with the disease control animal group (*p* < 0.01). Treatment of CFA significantly reduced the stair climbing activity of all animals from days 5 to 21 compared with the normal group demonstrating the induction of hyperalgesia (*p* < 0.0001). However, the group treated with GEF and diclofenac had improved scores from days 12 and 16, which were significantly increased until day 21. In addition, the motility score, which was reduced by CFA treatment, steadily and significantly recovered from days 16 to 21 in the GEF-treated group. This study indicated that GEF has anti-arthritic activity in an animal model of arthritis induced with CFA.

### 3.4. Anti-Inflammatory Effects

Inflammation is the immune system’s first biological response to infection, injury, or irritation [68]. There is evidence that anti-inflammatory effects are mediated through the modulation of various inflammatory cytokines [69]. Interleukin 6 (IL-6) is produced rapidly and transiently in response to infection and tissue damage and contributes to host defense through the stimulation of acute phase responses, as well as hematopoietic and immune responses [70]. A study conducted by Barve, K. [38] showed that, after 16 weeks, IL-6 levels in the plasma and thoracic aorta were significantly increased in induced hyperlipidemic animals compared with normal animals (*p* < 0.0001 and *p* < 0.001, respectively). In animals treated with 25 mg/kg GEF, IL-6 levels in the thoracic aorta were decreased but not significant, and IL-6 levels were significantly decreased in animals treated with 50 or 100 mg/kg GEF (*p* < 0.01). In addition, plasma IL-6 levels were significantly reduced in a dose-dependent manner by treatment with all concentrations of GEF (*p* < 0.0001). 

### 3.5. Antidepressant and Anxiolytic Effects

Depression and anxiety are the most common mental disorders and are the leading cause of psychosocial dysfunction [71]. Pharmacotherapy is the first line treatment of these disorders, but it can impose some problems, including sedation and amnesia, causing tolerance, psychomotor effects, and dependence [72]. These considerations emphasized the importance of finding new psychopharmacological drugs with an immediate onset of action and fewer side effects [73]. In this regard, natural products represent promising candidates for the pharmacological treatment of these pathologies [74].

Dhamija, I. et al. [40] investigated the antidepressant and anti-anxiety effects of *G. indica* using mouse and rat models in which the rind of *G. indica* was triturated with a mortar and pestle and mixed with feed. Elevated plus maze (EPM), light–dark model (LDM), and hole-board tests (HBT) were used to evaluate anxiolytic effects, and the forced swim test (FST), tail suspension test (TST), reserpine-induced hypothermia model, measurement of locomotor activity, and estimations of biochemical were used as methods to measure antidepressant activity. Positive controls received 1 mg/kg diazepam (standard anxiolytic), 15 mg/kg imipramine and 20 mg/kg fluoxetine (standard antidepressant agents), and 0.5, 1, and 2% *w*/*w* of *G. indica* rind mixed into their food. In the LDM, a significant increase in the length of time animals stayed in the lighted compartments indicated anxiolytic activity and the reverse of this represented anxiogenic activity. In the HBT models, head-dipping behavior reflected the animal’s emotional state. Administration of *G. indica* resulted in a significant increase in the length of time mice stayed in the light box in the LDM (*p* < 0.05). In addition, treatment with 0.5, 1, and 2% *w*/*w* of *G. indica* increased the number of head dips. In the EPM, anxiolytic effects were found in the state of open arms by increasing the length of stay and the number of entries. On the other hand, spending less time with arms outstretched reflected anxiety effect. *G. indica* at concentrations of 0.5, 1, and 2% *w*/*w* dose-dependently improved the time spent and number of entries in the open arms of the EPM (*p* < 0.05). Immobility refers to a condition in animals known as helplessness that can be converted by antidepressants such as fluoxetine and imipramine. A decrease in immobility indicates the absence of depression. *G. indica* at the concentrations tested in the FST and TST models significantly decreased animal immobility (*p* < 0.05). The rectal temperature in mouse using a reserpine-induced hypothermia model reflected the depletion of noradrenaline and serotonin in the brain, and 1% *w*/*w G. indica* significantly reversed the reserpine-induced hypothermia. *(p* < 0.05). These results indicate that *G. indica* fruit rind as a functional food exhibits significant antidepressant and anti-anxiety effects, without impairing motor activity and not involving CNS stimulation.

### 3.6. Antibacterial Activity

Bacterial resistance to antibiotics has rapidly increased and is one of the major threats to public health in the 21st century [75]. Natural products have historically been successful as a source of new antibiotics, and most existing classes of antibiotics are derived from natural compounds [76]. The potential for antibacterial effects of *G. indica* has been reported [77,78,79]. 

Sangaonkar, G.M. et al. [37] noted the antioxidant effect and the antibacterial effects of the biogenic synthesis of AgNPs using *G. indica* fruit extract. It was reported that AgNPs exhibit potential antibacterial activity against methicillin resistant *Staphylococcus aureus* (MRSA) by a mechanism different from that of antibiotics [80]. Based on this potential effect of AgNPs, they tested the antimicrobial activity 20 μg/μL AgNPs against 7 bacterial pathogens (*Escherichia coli, Bacillus subtilis, Staphylococcus aureus, Pseudomonas aeruginosa, Salmonella enterica typhi, Proteus vulgaris, Serratia marcescens*) and found that it inhibited the growth of *E. coli, B. subtilis, S. aureus*, and *P. aeruginosa*, with a clear region of inhibition in the range of 12–15 mm. However, *S. enterica typhi*, *P. vulgaris*, and *S. marcescens* were not inhibited by AgNP treatment. As a result of measuring the minimum inhibitory concentration using the micro-broth dilution method reported by Siddiq et al. [81], the inhibitory power of AgNPs was estimated to be 20 mg/mL for *E. coli*, *B. subtilis*, and *S. aureus*, and 40 mg/mL for *P. aeruginosa*. In addition, the minimum bactericidal concentration for *E. coli*, *B. subtilis*, and *S. aureus* was the same as the minimum inhibitory concentration, and 160 mg/mL for *P. aeruginosa*. These results suggest that AgNPs biosynthesized with *G. indica* fruit extract have important applications in biomedical fields as an antibacterial agent.

### 3.7. Hepatoprotective Activity

As mentioned above, Panda, V. et al. [36] determined the antioxidant effects of *G. indica* through various mechanisms. Significantly increased levels of serum enzymes such as aspartate aminotransferase (AST), alanine aminotransferase (ALT), and alkaline phosphatase were observed in an EtOH-treated animal group compared with the normal animal group (*p* < 0.001). These activities were due to the release of enzymes into the blood following extensive liver damage caused by the EtOH. Treatment with 400 or 800 mg/kg aqueous extracts of *G. indica* reduced AST activity (*p* < 0.001). However, ALT and AST activities were significantly improved only in the 800 mg/kg of the *G. indica*-treated group, similar to treatment with silymarin, which protects hepatocytes from toxins (*p* < 0.05). Moreover, increased serum triglyceride (sTG) levels and decreased total protein (TP) and albumin (Alb) levels were observed in EtOH-fed rats compared with the normal group. These effects suggested the decreased activity of lipase enzyme responsible for lipoprotein uptake by extrahepatic tissues. Treatment with aqueous extracts of *G. indica* restored these increased sTG levels and decreased TP levels in a dose-dependent manner. However, treatment with aqueous extracts of *G. indica* ameliorated Alb levels, although this did not reach statistical significance. Finally, light microscopy histopathological studies of liver sections fixed in 10% buffered formalin, embedded in paraffin, cut to 5 µm, and stained with hematoxylin and eosin (H/E), showed a moderate-to-marked degree of histoarchitectural changes in animals treated with EtOH. There were moderate-to-severe abscesses and bleeding, and the central vein revealed congestion of sinusoids. Moderate mononuclear inflammatory infiltration was observed in all zones, and many hepatocytes showed degenerative changes. However, the group treated with 400 mg/kg aqueous extracts of *G. indica* had mild-to-moderate mononuclear inflammatory infiltration in all areas with moderate fat changes and few regenerated cells. In the group treated with 800 mg/kg aqueous extracts of *G. indica*, slight or minimal fat changes were present around the dilated central vein, and many regenerative cells were observed. This histology was similar to that of the silymarin-treated group, a proven hepatoprotective, with a tendency to be more normal compared with the group treated with 400 mg/kg aqueous extracts of *G. indica*. These findings suggest that the hepatoprotective effect of aqueous extracts of *G. indica* in ethanol-induced hepatotoxicity may be due to increased endogenous antioxidant levels and the inhibition of lipid peroxidation in the liver.

It is important to note that there are concerns about the risk of hepatotoxicity related to garcinia food supplements from a recent report by the Scientific Committee of the Spanish Agency for Food Safety and Nutrition (AESAN) [82]. As a result of several previous studies [83,84,85], *G. gummi-gutta* (*G. cambogia*) and HCA, its main active ingredient, can cause herb-induced hepatotoxicity (HILI) including elevated liver enzyme levels, hepatitis, cholestasis, and jaundice. Furthermore, these acute liver damages are known to persist even after discontinuation of their intake. Although no research results have been reported on the direct association between *G. indica* and acute liver injury, more scientific and clinical investigations on the effect of *G. indica* on liver health since *G. indica* also contain HCA.

### 3.8. Cardioprotective Activity

Atherosclerosis is one of the leading causes of cardiovascular disease, with significant mortality and morbidity worldwide. Barve, K. [38] calculated various indicators to predict the risk of atherosclerosis and subsequent cardiovascular disease using lipid values as follows: atherogenic index of plasma (AIP) = (log (triglycerides/high-density cholesterol)), cardiac risk ratio (CRR) = (total cholesterol/high-density cholesterol), atherogenic coefficient (AC) = (total cholesterol–high-density cholesterol/high-density cholesterol). The values of AIP, CPR, and AC were significantly higher at the end of 12 weeks in the group of animals fed a modified Western diet compared with the group of normal animals (*p* < 0.01, *p* < 0.001 and *p* < 0.0001, respectively). However, treatment with all tested concentrations of GEF significantly reduced these values (*p* < 0.0001), almost normalizing the levels, and attenuating the risk of atherosclerosis. Histopathological studies using light microscopy were performed by staining tissue sections of the thoracic aorta of mice with H/E. Then, lesions were scored for global pathological changes (lesion scores) as follows: score 0 = no change, score 1 = minimal changes, score 2 = minor changes, score 3 = moderate changes, and score 4 = significant changes. In the disease-induced animals, histopathological analysis of the aorta showed thickening of the vessel wall, and the lesion score of 3.33. Treatment with 25 or 50 mg/kg GEF improved some of these changes, resulting in a lesion score of 2.33, but this was not statistically significant. On the other hand, treatment with simvastatin or 100 mg/kg GEF showed completely normal vasculature with lesion scores of 0.66 and 1 (*p* < 0.0001 and *p* < 0.001, respectively).

Patel et al. [39] evaluated the cardioprotective effects of aqueous extracts of *G. indica* by ISO-induced myocardial injury in Wistar albino rats. The aqueous extract of *G. indica* did not affect the levels of cardiac injury markers including serum troponin I, lactate dehydrogenase, creatinine kinase-MB, ALT, AST, and uric acid, which were significantly increased by ISO treatment. In addition, histopathological analysis demonstrated that the animals treated with aqueous *G. indica* extract had improved edema, infiltration, and necrosis levels, but no statistical significance was observed compared with the ISO control group. The results of these experiments suggest that aqueous extracts of *G. indica* do not have a cardioprotective effect against myocardial damage, and further studies with larger sample sizes and higher dose ranges may be needed to evaluate its cardioprotective effects.

## 4. Pharmacological Effects of Garcinol, a Major Active Constituent from *G. indica*

As mentioned above, various pharmacological activities of extracts and fractions of *G. indica* have been reported [33]. *G. indica* contains a major active compound called garcinol. This component is a yellow-colored, fat soluble pigment found in the rinds of *G. indica* at a level of 2–3%, which can be separated from the fruit rinds by EtOH and hexane extraction [35]. Garcinol shows powerful antioxidant activity, since it contains both phenolic hydroxyl groups and a β-diketone moiety, and in this respect, it resembles the structure of curcumin [48]. In addition, like the various pharmacological activities of *G. indica* mentioned above, there are many reported mechanisms by which garcinol also acts as an antioxidant, anti-inflammatory, or anticancer effects. It inhibited free-radical DPPH and was shown to have antioxidant activity on arachidonic acid metabolism and NO radical synthesis. These activities are involved in inflammation and carcinogenesis. Garcinol effectively inhibited inducible nitric oxide synthase (iNOS) synthesis by suppressing the activation of nuclear factor NFκB, and diminished the production of extracellular signal-regulated kinase 1/2, cyclooxygenase-2, and prostaglandins. In addition, it inhibited the activation of 5-lipoxygenase, which is responsible for the production of inflammatory molecules such as leukotrienes. These actions have inspired studies on inflammation-related cancers. In addition to reducing inflammation, the mechanisms underlying anticancer effects such as induction of apoptosis, inhibition of cell growth and proliferation, stimulation of cell cycle arrest and prevention of cancer cell metastasis have been identified. Consequently, numerous preclinical studies have reviewed the antitumor potential of garcinol in a variety of oncological variants, including colon, breast, prostate, head, and neck cancer, and hepatocellular carcinoma [86,87]. Furthermore, the antioxidant and anti-inflammatory properties of garcinol could be extended to a neuroprotective role, and garcinol acts as a potent natural HAT inhibitor, and has presented promising results in molecular interactions studies against MAO-B and catechol-O-methyltransferase, as well as in l-DOPA-induced dyskinesia. It has recently been reported the ability of garcinol to modulate memory and cognition by affecting nerve growth and survival and altering the neurochemical state of the brain [88].

## 5. Conclusions

*G. indica* has various pharmacological activities including antioxidant, anti-obesity, anti-arthritis, anti-inflammatory, antibacterial, hepatoprotective, cardioprotective, anti-depressant and anti-anxiety effects. These characteristics are consistent with the previously reported activity of abundant phytochemical components such as garcinol, HCA, cyanidin-3-sambubioside and cyanidin-3-glucoside isolated from *G. indica*. These studies suggest the potential of *G. indica* as a promising therapeutic agent for controlling and preventing various diseases. However, given that most of the trials have been conducted either in vitro or in vivo, further study at the clinical level is needed to establish the efficacy and safety of *G. indica* in humans.

## Figures and Tables

**Table 1:** Summary of pharmacological studies for *Garcinia indica*.

Pharmacological Activity	Tested Substance	In Vitro/ In Vivo	Model	Dose/Concentration	Ref.
Antioxidant	Aqueous extract	In vivo	Wister albino rats	400 and 800 mg/kg	[36]
	Fruit extract	In vitro	-	1.5 mM	[37]
	Garcinol-enriched fraction	In vivo	C57BL/6 male mice	25, 50, and 100 mg/kg	[38]
	Aqueous extract	In vitro	-	-	[39]
		In vivo	Wister albino rats	250 and 500 mg/kg	
	*G. indica* fruit rind powder	In vivo	Swiss albino mice, Wister rats	0.5, 1, and 2% *w*/*w*	[40]
Anti-obesity	Garcinol-enriched fraction	In vivo	C57BL/6 male mice	25, 50, and 100 mg/kg	[38]
	Fruit extract	In vitro	3T3-L1 preadipocytes	1 and 2 µg/mL	[41]
		In vivo	C57BL/6 male mice	0.01% *w*/*w*	
Anti-arthritic	Garcinol-enriched fraction	In vivo	Male Wistar rats	10 mg/kg	[42]
Anti-inflammatory	Garcinol-enriched fraction	In vivo	C57BL/6 male mice	25, 50, and 100 mg/kg	[38]
Antidepressant and anxiolytic effect	*G. indica* fruit rind powder	In vivo	Swiss albino mice, Wister rats	0.5, 1, and 2%	[40]
Antibacterial	Fruit extract	In vitro	-	1.5 mM	[37]
Hepatoprotective	Aqueous extract	In vivo	Wister albino rats	400 and 800 mg/kg	[36]
Cardioprotective	Garcinol-enriched fraction	In vivo	C57BL/6 male mice	25, 50, and 100 mg/kg	[38]
	Aqueous extract	In vivo	Wister albino rats	250 and 500 mg/kg	[39]

## Data Availability

Data are contained within the article.
